# Pseudogene *BMI1P1* expression as a novel predictor for acute myeloid leukemia development and prognosis

**DOI:** 10.18632/oncotarget.10156

**Published:** 2016-06-18

**Authors:** Ling-Yu Zhou, Ling-Ling Zhai, Jia-Yu Yin, Minse Evola-Deniz Vanessa, Jiao Zhou, Jing Zhang, Xi Tang, Jiang Lin, Jun Qian, Zhao-Qun Deng

**Affiliations:** ^1^ Department of Laboratory Center, The Affiliated People's Hospital of Jiangsu University, Zhenjiang, 212002, Jiangsu, People's Republic of China; ^2^ Department of Hematology, The Affiliated People's Hospital of Jiangsu University, Zhenjiang, 212002, Jiangsu, People's Republic of China

**Keywords:** pseudogene, acute myeloid leukemia, BMI1P1, tumor marker

## Abstract

The *BMI1P1* levels of 144 de novo AML patients and 36 healthy donors were detected by real-time quantitative PCR (RQ-PCR). *BMI1P1* was significantly down-regulated in AML compared with control (*P* < 0.001). A receiver operating characteristic (ROC) curve revealed that *BMI1P1* expression could differentiate patients with AML from control subjects (AUC = 0.895, 95% CI: 0.835–0.954, *P* < 0.001). The percentage of blasts in bone marrow (BM) was significantly lower in *BMI1P1* high-expressed group versus low-expressed group (*P* = 0.008). *BMI1P1* high-expressed cases had significantly higher complete remission (CR) than *BMI1P1* low-expressed cases (*P* = 0.023). Furthermore, Kaplan–Meier demonstrated that both whole AML cohort and non-M3-AML patients with low *BMI1P1* expression showed shorter leukemia free survival (LFS, *P* = 0.002 and *P* = 0.01, respectively) and overall survival (OS, *P* < 0.001 and *P* = 0.011, respectively) than those with high *BMI1P1* expression. Multivariate analysis also showed that *BMI1P1* over-expression was an independent favorable prognostic factor for OS in both whole and non-M3 cohort of AML patients (HR = 0.462, 95% CI = 0.243–0.879, *P* = 0.019 and HR = 0.483, 95% CI = 0.254–0.919, *P* = 0.027). To further investigate the significance of *BMI1P1* expression in the follow-up of AML patients, we monitored the *BMI1P1* level in 26 de novo AML patients and found that the *BMI1P1* level increased significantly from the initial diagnosis to post-CR (*P* < 0.001). These results indicated that *BMI1P1* might contribute to the diagnosis of AML and the assessment of therapeutic effect.

## INTRODUCTION

Acute myeloid leukemia (AML) is the most common type of myeloid leukemia characterized by uncontrollable heterogeneous clonal disorder and accumulation of malignant haemopoietic progenitor cells in bone marrow and blood [1]. Currently, cytogenetics, molecular genetics and clinical studies, which are associated with pathogenesis of AML, provide useful guides for identifying patients' prognosis information and better approaches to therapy [2, 3]. Identifying molecular markers contributes to differentiating patients' risk and refining the prognosis of patients with AML [4].

Recently, significant attention has been paid to non-coding RNAs (*ncRNAs*), including microRNAs, long non-coding RNAs (*LncRNAs*), small interfering RNAs (*siRNAs*), pseudogenes, etc. [5]. It is being increasingly clear that *ncRNAs* play a functional role in diverse cellular processes, with their dysregulation already associated with origination and progression of cancers [6]. Pseudogenes were initially defined as unnecessary copies of coding genes by the fact that they lost the ability of coding functional protein due to gene mutations, a lack of transcription, or their inability to encode RNA [7]. Nowadays, accumulating evidence reveals that pseudogenes are associated with various diseases and functions, one of which is cancer development [7–9]. Pseudogenes may be strongly linked to oncogenic development and can be used as diagnostic and prognostic biomarkers in different human cancers [10]. Patients with gastric cancer (GC) are characterized by lower serum levels of *PTENP1* pseudogene, which shows a diagnostic ability (AUC > 0.8) when compared with healthy controls [11]. Over-expression of *SUMO1P3* pseudogene has also shown its ability for discriminating GC patients from patients with benign gastric disease [12], and its over-expression was also positively correlated with the state of bladder cancer [13]. Analogously, pseudogene *INTS6P1* expression is high and steady in normal people compared with hepatocellular carcinoma (HCC) patients. Thepseudogene diagnostic value may be equal to that of alpha-fetal protein (AFP), the most common biomarker used in the diagnosis of HCC [14]. Besides being accurate diagnostic markers, pseudogenes also can be used as valuable prognostic markers to stratify cancer patients. For example, Hayashi et al. [15] showed that over-expressed *OCT4-pg1* combined with genomic amplification like c-MYC can promote tumor cells' proliferation and angiogenesis while inhibiting apoptosis. *OCT4-pg1* amplification was positively correlated with associated with a decreased overall survival in gastric cancer. As another example, the pseudogene *PTENP1* affected the post-transcriptional regulation of its parental gene (*PTEN*) through competition for *PTEN*-targeting *miRNAs*, and patients who did express *PTENP1* showed a more favorable outcome compared to those who did not express *PTENP1* in clear cell renal cell carcinoma [16]. Previous works strongly suggested that pseudogenes did not only help us to understand the cancer pathogenesis but also could serve as a new panel of useful biomarkers for cancers. Until now, several pseudogenes have been identified in normal and malignant hematopoietic cell [17, 18], but the function and the regulatory mechanisms of these pseudogenes for AML have not been defined in any studies yet.

BMI1 (Moloney murine leukemia virus integration site 1) is a polycomb ring finger oncogene involved in the regulation of p16 and p19, which are inhibitor genes for cell cycle progression [19]. Its expression plays a critical role in several signaling including wnt, akt, notch, hedgehog and receptor tyrosine kinase (RTK) pathway [20]. *BMI1* is essential for efficient self-renewing and reconstituting activity of hematopoietic stem cells as well as leukemic stem cells and neural progenitors [21, 22]. Over-expression of *BMI1* has been reported in a number of human malignancies, such as bladder, skin, prostate, breast, ovarian, colorectal as well as hematopoietic malignances [23], and its over-expression is associated with poor prognostic in these malignancies. *BMI1* pseudogene, namely *BMI1P1*, located on human chromosomal band Xq12, which has high homology with *BMI1*, has barely been studied in any cancers. This study was aimed to investigate the *BMI1P1* expression in de novo AML patients and to analyze its clinical relevance, whether it might serve as a biomarker for predicting disease prognostic.

## RESULTS

### *BMI1P1* expression in normal controls and AML patients

**Figure 1:**
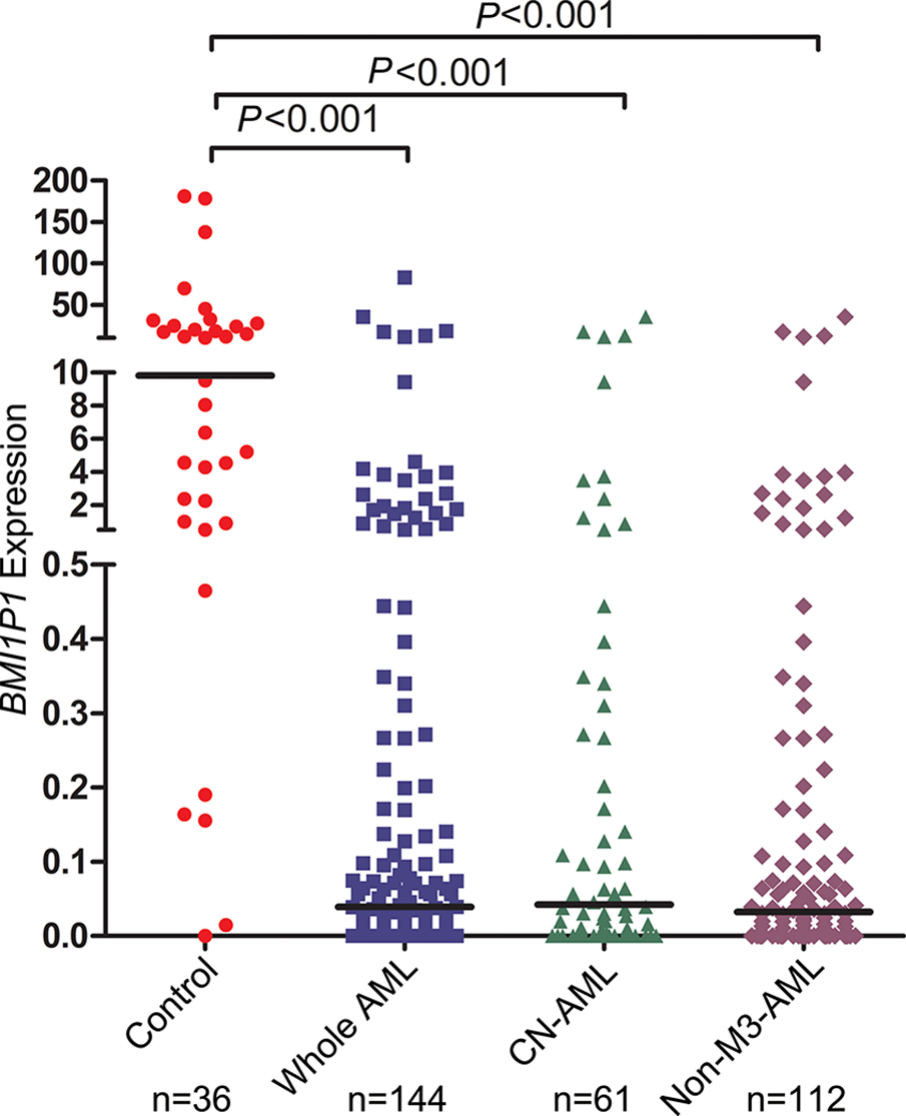
Relative expression levels of *BMI1P1* in AML and controls Expression of *BMI1P1* in BMNCs was measured via using RQ-PCR in healthy controls, whole AML, CN-AML and non-M3-AML samples. Horizontal lines represent the median, and each dot represents an individual sample. Statistical analysis was performed using Wilcoxon tests, and significance was defined as *P* < 0.05.

**Figure 2:**
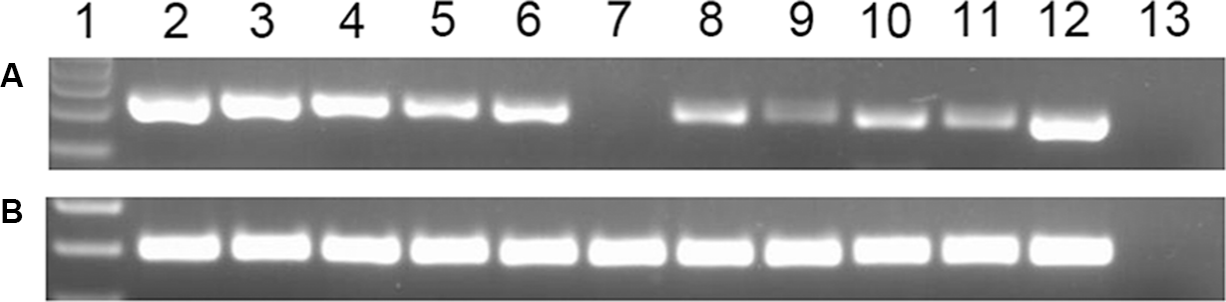
Electrophoresis results of RQ-PCR products in AML patients lane 1: Gene RulerTM 100 bp DNA ladder; lane 2–3: The representative electrophoresis results of RQ-PCR products, which were randomly selected from 36 healthy controls, were loaded on lane 2–3; lane 4–11: The representative electrophoresis results of RQ-PCR products, which were randomly selected from 144 AML patients, were loaded on lane 4–11; lane 12: The cloned plasmid carrying *BMI1P1* cDNA was constructed as positive control and the result was loaded on lane 12.; lane13: negative control. (**A**) *BMI1P1*; (**B**) *ABL*.

In our experiment, the *BMI1P1* mRNA level in normal controls ranges from 0.000 to 660.68 with a median level of 9.825. The level of *BMI1P1* expression in AML cases (0–83.090, median 0.039) appears significantly down-regulated than control subjects (*P* < 0.001, Figure 1). In addition, down-regulated level of *BMI1P1* expression, which is compared with its level in control subjects (*P* < 0.05 for each subtype, Table 1), was found in different AML subtypes. The typical electrophoresis results of RQ-PCR products are shown in Figure 2.

**Table 1:** BMI1P1 expression level in different AML subtypes

Groups	Subtypes and stages	Subjects		*BMI1P1* expression		*P*
		Number	%	Median	Range	
Total AML	Total	144	100	0.039	0–83.090	< 0.001
*N* = 144	FAB					
	M0	1	0.7	0.000	—	—
	M1	10	6.9	0.025	0–3.491	< 0.001
	M2	52	36.1	0.040	0–12.191	< 0.001
	M3	32	22.2	0.079	0–83.092	< 0.001
	M4	29	20.1	0.030	0–35.430	< 0.001
	M5	15	10.4	0.016	0–2.697	< 0.001
	M6	5	3.5	0.169	0–17.308	0.018
WHO						
	AML with t(8;21)	9	6.3	0.031	0–3.833	< 0.001
	APL with t(15;17)	30	20.8	0.079	0–83.092	< 0.001
	AML with 11q23 translocation	1	0.7	0.013	—	—
	AML without maturation	9	6.3	0.021	0–3.491	< 0.001
	AML with maturation	42	29.2	0.046	0–12.791	< 0.001
	Acute myelomonocytic leukemia	29	20.1	0.030	0–35.430	< 0.001
	Acute monoblastic and monocytic leukemia	13	9.0	0.036	0–2.697	< 0.001
	Acute erythroid leukemia	4	2.8	0.101	0–17.308	0.046
Control		36	100	9.825	0–660.68	—

*P*: significance versus control.

### Differentiating value of *BMI1P1* expression

**Figure 3:**
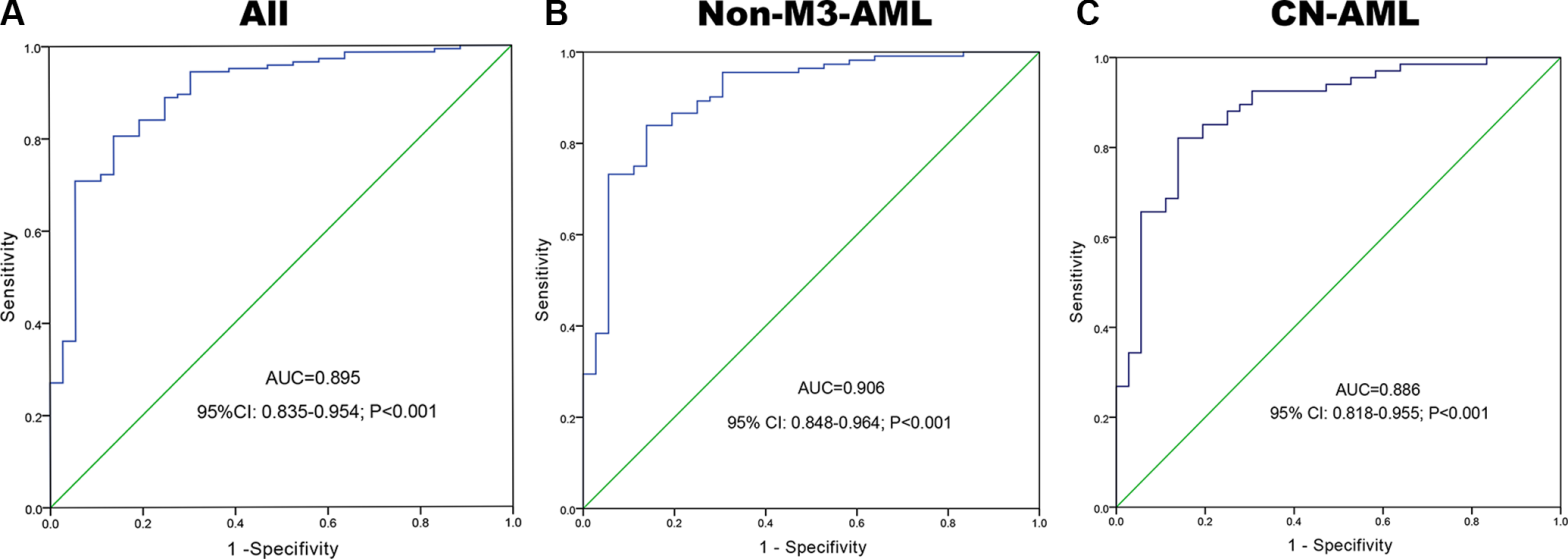
*BMI1P1* expression offers a powerful diagnostic tool in identification of AML patients (**A**) All patients; (**B**) non-M3-AML; (**C**) CN-AML. ROC analysis showed that the area under the curve (AUC) of *BMI1P*1 was 0.895 (*P* < 0.001), 0.906 (*P* < 0.001) and 0,886 (*P* < 0.001) in whole AML, non-M3-AML and CN-AML, respectively.

A receiver operating characteristic curve was constructed to analyze the diagnostic accuracy of *BMI1P1* expression. It revealed that *BMI1P1* expression could serve as a valuable biomarker for distinguishing whole AML patients from control subjects (AUC = 0.895, 95% CI: 0.835–0.954, *P* < 0.001) (Figure 3A). At the cut-off value of 0.159, the sensitivity and the specificity were 71% and 92%, respectively. Moreover, the level of *BMI1P1* expression might also function as a valuable biomarker in non-M3 AML (AUC = 0.906, 95% CI: 0.848–0.964, *P* < 0.001) (Figure 3B) and CN-AML (AUC = 0.886, 95% CI: 0.818–0.955, *P* < 0.001) (Figure 3C) according to ROC curves analysis.

### Clinical and laboratory characteristics of AML

This cohort of 144 AML patients was divided into low-expressed group (< 0.159) and high-expressed group (≥ 0.159) according to the cut off value of 0.159. Age, white blood cells (WBC), hemoglobin (HB), platelets (PLT), FAB or WHO classifications and karyotypes did not differ significantly between *BMI1P1* low-expressed group and high-expressed group. We further investigated whether the level of *BMI1P1* was associated with patients' gene mutations. To test this hypothesis, we detected several gene mutations, such as *C/EBPA*, *NPM1*, *FLT3 ITD*, *C-KIT*, *IDH1/2*, *DNMT3A* and *U2AF1*. But we failed to find a significant correlation of gene mutations with *BMI1P1* in these patients (data not shown). However, the rate of over-expression of *BMI1P1* in female patients was significantly higher than that in male patients (*P* = 0.043). Also, the percentage of blasts in bone marrow (BM) was significantly lower in *BMI1P1* high-expressed group versus low-expressed group (*P* = 0.008). *BMI1P1* high-expressed cases had significantly higher complete remission (CR) than low-expressed cases (*P* = 0.023) (Table 2).

**Table 2:** Correlation between BMI1P1 expression and patients parameters

Patient's parameters	Status of *BMI1P1* expression		
	Low (*n* = 102)	High (*n* = 42)	*P*
Sex, male/female	63/39	18/24	0.043
Median age, years (range)	55.5 (10–93)	54.5 (15–85)	0.919
Median hemoglobin, g/L (range)	75.0 (34–142)	74 (32–119)	0.916
Median WBC, ×10^9^/L (range)	17.7 (0.8–528.0)	8.2 (0.3–203.6)	0.131
Median platelets, ×10^9^/L (range)	36.0 (3–447)	47.5 (4–190)	0.351
BM blasts, % (range)	48.5 (3–97.5)	28.0 (1–94)	0.008
FAB			0.339
M0	1 (1%)	0 (0%)	
M1	6 (6%)	4 (10%)	
M2	38 (37%)	14 (33%)	
M3	20 (20%)	12 (29%)	
M4	22 (22%)	7 (17%)	
M5	13 (13%)	2 (5%)	
M6	2 (2%)	2 (5%)	
WHO			0.800
AML with t(8;21)	7 (7%)	2 (5%)	
APL with t(15;17)	19 (19%)	11 (26%)	
AML with 11q23 translocation	1 (1%)	0 (0%)	
AML without maturation	6 (6%)	3 (7%)	
AML with maturation	30 (29%)	12 (29%)	
Acute myelomonocytic leukemia	22 (22%)	7 (17%)	
Acute monoblastic and monocytic leukemia	11 (11%)	2 (5%)	
Acute erythroid leukemia	2 (2%)	2 (5%)	
No data	4 (4%)	3 (7%)	
Karyotype classification			0.707
Favorable	25 (25%)	13 (31%)	
Intermediate	55 (54%)	22 (52%)	
Poor	13 (13%)	4 (10%)	
No data	9 (9%)	3 (7%)	
Karyotype			0.518
normal	41 (40%)	20 (48%)	
t(8;21)	7 (7%)	2 (5%)	
t(15;17)	19 (19%)	11 (26%)	
11q23	1 (1%)	0 (0%)	
complex	11 (11%)	4 (10%)	
others	15 (15%)	2 (5%)	
No data	8 (8%)	3 (7%)	
Gene Mutation*			
*C/EBPA* (+/−)	10/82	7/28	0.242
* NPM1* (+/−)	11/81	3/32	0.756
* FLT3 ITD* (+/−)	15/77	1/34	0.068
* C-KIT* (+/−)	3/89	0/35	0.561
CR(+/−)	34/54	21/12	0.023
*BMI1P1* transcript	0.01 (0–0.14)	1.48 (0.17–83.09)	< 0.001

WBC, white blood cells; FAB, French-American-British classification; AML, acute myeloid leukaemia; CR, complete remission;

* percentage was equal to the number of mutated patients divided by total cases in each group.

### Correlation between *BMI1P1* expression and clinical outcome

**Figure 4:**
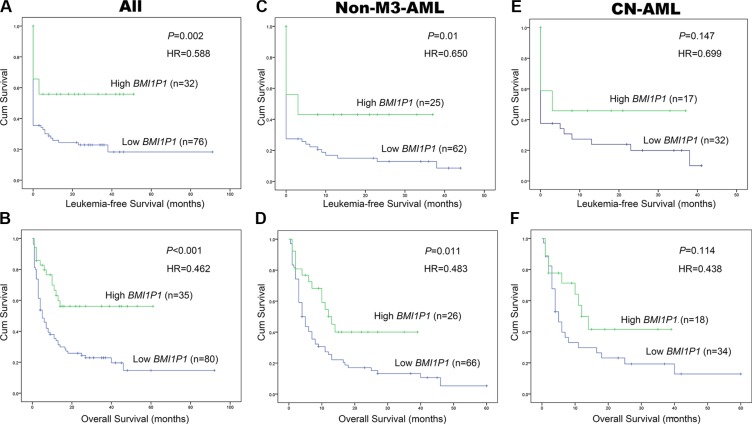
High level of *BMI1P1* predicts favorable prognosis in AML (**A**) LFS were investigated for whole AML patients according to expression of *BMI1P1.* (**B**) OS was investigated for whole AML patients according to expression of *BMI1P1*. (**C**) LFS was investigated for non-M3-AML patients according to expression of *BMI1P1*. (**D**) OS was investigated for non-M3-AML patients according to expression of *BMI1P1.* (**E**) LFS was investigated for CN-AML patients according to expression of *BMI1P1*. (**F**) OS was investigated for CN-AML patients according to expression of *BMI1P1*. Survival analysis was performed via Kaplan–Meier survival analysis, with differences between curves analyzed via a log-rank test. Significance was defined as *P* < 0. 05.

**Figure 5:**
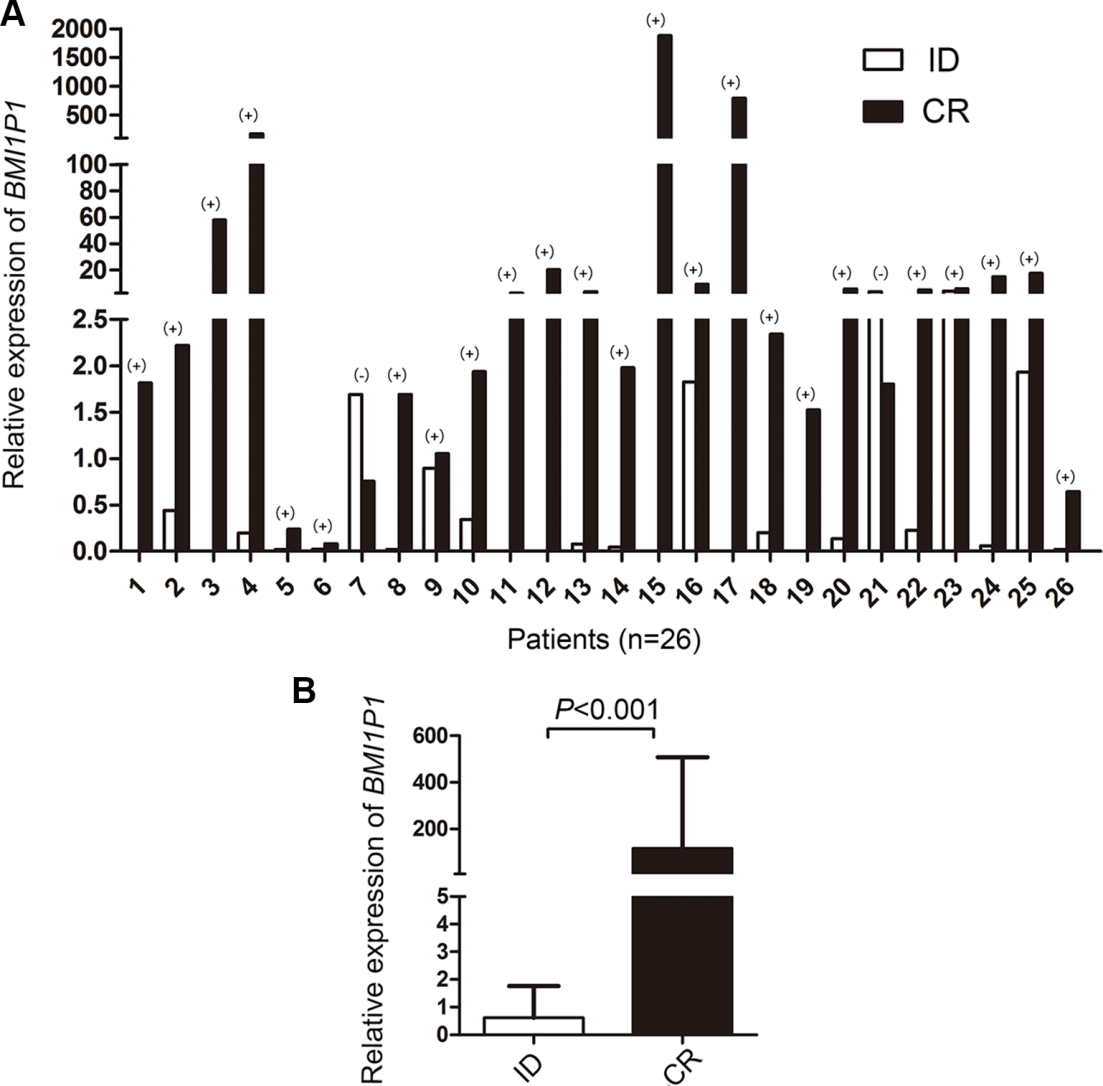
Changes of *BMI1P1* expression in 26 AML patients (**A**) The differential *BMI1P1* levels in AML patients (*n* = 26) were measured by RQ-PCR from the initial diagnosis to complete remission. (+) and (−) indicates up-regulation and down-regulation, respectively. (**B**) *BMI1P1* was up-regulated in 92% (24/26) of post-CR versus ID (*P* < 0.001), the statistical significance was found by using Wilcoxon tests. Significance was defined as *P* < 0.05.

115 AML patients with mean follow-up time of 7 months (range, 1–92 months) were included in survival analysis. Our research showed that the high level of *BMI1P1* exhibited a positive impact on patients' survival. Kaplan–Meier demonstrated that patients with low-expressed *BMI1P1* had significantly shorter leukemia free survival (LFS, median 0 vs 6.5 months, respectively, *P* = 0.002) and overall survival (OS, median 5 vs 13 months, respectively, *P* < 0.001) than *BMI1P1* high-expressed patients in the whole cohort of AML patients (Figure 4A, 4B). This favorable prognosis associated with *BMI1P1* over-expression was also observed in the non-M3 cohort of AML patients (LFS, median 0 vs 3 months, respectively, *P* = 0.01; OS, median 10.5 vs 4 months, respectively, *P* = 0.011) (Figure 4C, 4D). However, we did not find that LFS and OS were obviously altered in the CN-AML group (Figure 4E, 4F). Multivariate analysis, applying age (≤ 60 y vs > 60 y), sex (male vs female), WBC (≥ 30 × 10^9^/L vs < 30 × 10^9^/L), HB (< 110 g/L vs ≥110 g/L), PLT (100×10^9^/L vs 100 × 10^9^/L), karyotype classifications (favorable vs intermediate vs poor), gene mutations (mutant vs wild-type) and *BMI1P1* expression status (high vs low) as covariates, also showed that *BMI1P1* over-expression was an independent favorable prognostic factor for OS in both whole and non-M3 cohort of AML patients (HR = 0.462, 95% CI = 0.243–0.879, *P* = 0.019 and HR = 0.483, 95% CI = 0.254–0.919, *P* = 0.027, Table 3). However, we failed to find that *BMI1P1* was an independent favorable prognostic factor for LFS in the two above groups (data not shown). To further investigated whether levels of *BMI1P1* factored in patients' response to therapy, we monitored *BMI1P1* levels of 26 patients with AML from the initial diagnosis to complete remission (Figure 5A). As we expected, the levels of *BMI1P1* increased significantly from initial diagnosis to the post-CR (*P* < 0.001) (Figure 5B).

**Table 3:** Multivariate analyses of prognostic factors for overall survival in whole AML and non-M3 AML cases

Covariates	whole AML		non-M3 AML	
	Hazard ratio (95% CI)	*P*	Hazard ratio (95% CI)	*P*
Sex	1.246 (0.688–2.257)	0.467	1.360 (0.764–2.423)	0.296
Age	1.346 (0.724–2.501)	0.347	1.205 (0.614–2.364)	0.588
WBC	1.591 (0.966–2.623)	0.068	1.406 (0.839–2.358)	0.196
HB	0.889 (0.355–2.225)	0.801	0.862 (0.323–2.305)	0.768
PLT	1.132 (0.535–2.393)	0.746	1.159 (0.551–2.439)	0.697
Karyotype classifications	4.049 (1.942–8.439)	0.000	3.119 (1.113–8.738)	0.030
*BMI1P1* expression	0.462 (0.243–0.879)	0.019	0.483 (0.254–0.919)	0.027
*FLT3* mutation	0.645 (0.299–1.393)	0.265	0.654 (0.263–1.625)	0.360
*NPM1* mutation	1.967 (0.837–4.622)	0.121	1.473 (0.665–3.261)	0.340
*C/EBPA* mutation	0.634 (0.274–1.468)	0.287	0.665 (0.280–1.582)	0.357
*C-KIT* mutation	1.876 (0.243–14.462)	0.546	4.037 (0.519–31.406)	0.183
*IDH1* and *IDH2* mutation	7.663 (2.177–26.982)	0.002	10.512 (2.914–37.916)	0.000
*DNMT3A* mutation	0.730 (0.276–1.930)	0.526	0.783 (0.290–2.111)	0.629
*U2AF1* mutation	1.727 (0.510–5.848)	0.380	2.334 (0.702–7.758)	0.166

## DISCUSSION

Standard chemotherapy and hematopoietic stem cell transplantation are common therapeutic protocols for patients with AML. Approximately 90% of both t (8; 21) and inv (16) AML patients achieve a complete remission by accepting anthracycline- and cytarabine-based induction chemotherapy [24]. However, these therapeutic protocols on the elderly population or some special subtypes of AML are less well defined. In the present, personalized medicine in cancer treatment is favored and admired progressively. Patients who harbor different variation of the human genome in the cancer can be treated accordingly. A more detailed classification of the cancer genome and epigenome, thus, needs to be achieved in AML. To this end, karyotypes are frequently referred to as an essential tool for the recognition of distinct subtypes of AML and have helped to identify prognostic group. What is more, molecular markers like *FLT3*, *C/EBPA*, and *NPM1* gene mutations also show strong correlation with prognosis as well as some common molecular lesions, such as DNA methyltransferase 3 alpha (*DNMT3A*) and isocitrate dehydrogenase 1/2 (*IDH1/IDH2*) [25, 26]. However, a classification solely based on karyotypes and pathological features has shown its limitations, and there are less than 30% AML patients owning gene mutations [27]. Similarly, our findings on gene mutations agree with this point, for the percentage of gene mutations including *C/EBPA*, *NPM1*, *FLT3-ITD*, *C-KIT*, *IDH1/2*, *DNMT3A* and *U2AF1* was 13.4%, 11.0%, 12.6%, 2.4%, 5.6%, 7.9% and 3.9% in these patients, respectively. Therefore, more useful biomarkers are needed in clinical practices to divide this heterogeneous cohort of AML patients into multiple subtypes and offer guidance and evaluation in the treatment of each patient. Pseudogenes, which are highly homologous with their parental genes, are ideal candidates to sustain the expression of their parental genes by serving as competing endogenous RNAs (*ceRNAs*) which compete for the binding site of the same mRNAs [16, 28]. In addition, some could regulate the expression of functional genes by producing endogenous small interference RNAs (*siRNAs*) [29, 30] and antisense RNAs (*asRNAs*) [31, 32], and some even could encode functional proteins [33, 34]. It is speculated that pseudogenes can be the supplement to their parental genes via gene mutation in a particular position. Aberrant expression of pseudogenes can be used as diagnostic and prognostic biomarkers in human cancers [14–16]. In some cases, it has shown its higher diagnostic and prognostic trend than microRNAs and mRNAs [35]. Nevertheless, the expression levels and functions of pseudogenes in AML have been less studied.

*BMI1*(the parental gene of *BMI1P1*), a stem cell factor, was observed to be highly expressed in various types of human cancers [23, 36], including AML [37]. It was reported that *BMI1* was essential for leukemic reprogramming of myeloid progenitor cells (BM blasts) into leukemic stem cells [38] and played a crucial role in regulating the proliferative activity of leukemic stem and progenitor cells [21]. In this study, *BMI1P1* was found to be significantly down-regulated in de novo AML compared with healthy controls. This down-regulated level of *BMI1P1* was also observed in different AML subtypes. To our knowledge, this is the first report about *BMI1P1* expression in cancers. Our results also indicated that low *BMI1P1* expression might be a prospective biomarker for screening AML, especially CN-AML and non-M3-AML from healthy controls by ROC curves analysis. Furthermore, our results indicated that patients with lower *BMI1P1* expression had significantly higher BM blasts when compared with those with higher *BMI1P1*. *BMI1P1* may be involved in the negatively regulation of *BMI1* and leads to a decline of BM blasts in turn. More researches are needed to confirm this conjecture.

Our study further demonstrated that *BMI1P1* high-expressed patients achieved significantly better OS, LFS and CR in both the entire AML cohort and non-M3-AML patients. We also revealed that the expression of *BMI1P1* was an independent prognostic factor for OS in both whole and non-M3 cohort of AML patients according to multivariate analyses. As prognosis guides therapy, *BMI1P1* may be a future therapeutic target. As we know, assessment of gene mutations in AML contributes to identifying subgroups with markedly superior outcome (e.g, mutant *N* PM1 [39] or *C/EBPA* [40]) and inferior outcome (e.g, mutant *C-KIT* [41], *DNMT3A* [27], *FLT3 ITD* [42], *MLL/KMT2A* [27] or *WT1* [43]). To determine whether *BMI1P1* correlates with gene mutations in patients with AML, we tested 7 kinds of these gene mutations. However, the differences in the impact of mutations of *FLT3*, *NPM1*, *C/EBPA*, *C-KIT* on outcome were not found, and we also failed to find a significant correlation of gene mutations with *BMI1P1* in these patients. Interestingly, dynamic monitoring *BMI1P1* level in 26 cases of patients revealed that *BMI1P1* levels were significantly increased from the initial diagnosis to complete remission by mentioned therapeutic protocols. From the results above, we concluded that determination of *BMI1P1* levels could be used as an important indicator of disease prognosis and evaluation of curative effect. Obviously, prospective studies on larger series of AML patients are needed to confirm and expand our findings.

Unfortunately, limited information is available to describe the function of *BMI1P1*, which has never been reported as a tumor suppressor in any human cancer. However, we showed that AML patients with a high *BMI1P1* expression have a favorable outcome, suggesting that pseudogene *BMI1P1* might be a tumor suppressor. Pseudogene transcripts can serve as competing endogenous RNAs (*ceRNAs*) to regulate its parental coding genes' expression [44]. Because of their striking sequence homology, pseudogenes are the sequences that share multiple microRNA responsive elements (MREs) with their parental genes and that can compete with their parental coding genes for the binding site of shared microRNA molecules [10, 44]. Taken all together, *BMI1P1* may be functional by mediating miRNA expression in AML. Over-expression of *BMI1P1* transcripts may be expected to arrest the functions of oncomiRs targeting essential genes to cellular repression, through competitive binding to the oncomiRs and somehow resulting in suppression of AML. The next step is to design more additional studies, including *in vitro* and *in vivo* functional assays, stem cell-associated assays and the relationship between *BMI1P1* and its parental coding gene, to assess mechanisms for potentially effects of pseudogene *BMI1P1* for AML. In the future, prospective screening for *BMI1P1* expression and *BMI1P1*-targeted intervention may shed new light on the classification and treatment of AML.

In conclusion, our study showed that pseudogene *BMI1P1* was down-expressed in AML. Pseudogene *BMI1P1* may serve a biomarker for detection of AML. Interestingly, *BMI1P1* may serve as an important prognostic and initial treatment marker for AML.

## MATERIALS AND METHODS

### Patients and samples

The bone marrows collected from 180 samples, including 144 patients with de novo AML treated in the Affiliated People' Hospital of Jiangsu University and 36 healthy donors regarded as normal controls after obtaining the written informed consent. All the patients were standardly diagnosed according to the French-America-British (FAB) and the World Health Organization (WHO) criteria [45, 46]. Treatment protocol was described in our previously reported work [47]. The main clinical and laboratory characteristics of the patient cohort were summarized in Table 1.

### RNA isolation, reverse transcription and real-time quantitative PCR

Mononuclear cells from bone marrow samples were separated by Ficoll-Hypaque gradient. Total RNA from bone marrow mononuclear cells (BMNCs) was isolated by using Trizol reagent (Invitrogen, Carlsbad, CA, USA) according to the manufacturer's instructions. Reverse transcription was performed on iCycler Thermal Cycler (Eppendorf, Hamburg, Germany) using reaction mixture containing 2 μg of total RNA, dNTPs 10 mM, random hexamers 10 μM, RNAsin 80 units, and 200 units of MMLV reverse transcriptase (MBI Fermentas, Hanover, USA) to synthesize cDNA. The system of reverse transcription was incubated for 10 min at 25°C, 60 min at 42°C, and then stored at −20°C.

*BMI1P1* was amplified using the primers 5′-AGTGGTATCTGCTCACT-3′ (forward) and 5′-CCTCC ACAAAGCACACACAT-3′ (reverse) with expected products of 210 bp. Real-time quantitative PCR (RQ-PCR) reactions were performed on a 7500 Thermocycler (Applied Biosystems, CA, USA). Reactions mixture of 20 μL in each tube consisting of 0.25 μM of primers, 10 μL SYBR Premix Ex Taq II, 0.4 μL 50×ROX (TaKaRa, Japan) and 50 ng of cDNA. RQ-PCR was carried out at 95°C for 30 s, followed by 40 cycles at 95°C for 5 s, 63°C for 30 s, 72°C for 30 s, and 80°C for 30 s to collect fluorescence, finally followed by the melting program at 95°C for 15 s, 60°C for 60 s, 99°C for 15 s, and 60°C for 15 s. Negative and positive controls were involved in all assays. The abundance of *BMI1P1* mRNA was estimated by housekeeping gene *ABL* (non-receptor tyrosine kinase). Relative levels of *BMI1P1* expression were calculated according to the following equation: N_BMI1P1_ = (E_BMI1P1_) ^ΔCT BMI1P 1(control-sample)^ ÷ (E_ABL_) ^ΔCT ABL (control-sample)^ ×1000‰. The parameter efficiency (E) derived from the formula E=10^(−1/slope)^ (the slope referred to CT versus cDNA concentration plot).

### Gene mutation detection

*IDH1/2*, *DNMT3A* and *U2AF1* mutations were detected according to the literatures reported previously [48–51]. The detection of nucleophosmin (*NPM1*) and *C-KIT* mutations was performed by using PCR and high-resolution melting analysis (HRMA). All positive samples were confirmed by direct DNA sequencing. *FLT3-ITD* and *C/EBPA* were detected by direct DNA sequencing.

### Statistical analysis

Statistical analyses were performed using the SPSS 18.0 software package (SPSS, Chicago, IL). Chi square test or Fisher exact test was used to compare the difference of qualitative data between patients groups. For comparison of quantitative data between groups; Kruskal-Wallis test (multiple groups) and Mann-Whitney *U*-test (two groups) were used. Receiver operating characteristic (ROC) curve and area under the ROC curve (AUC) were designed to assess the diagnostic value of *BMI1P1* expression in discriminating AML patients from normal controls. Kaplan–Meier test and Cox regression analysis were applied to analyze the impact of *BMI1P1* level on the prediction of survival in AML cases. For all analyses, a *P* value less than 0.05 (two-tail) was considered statistically significant.
